# Protocol for the Isolation of *Stratum Corneum* from Pig Ear Skin: Evaluation of the Trypsin Digestion Conditions

**DOI:** 10.3390/mps4040080

**Published:** 2021-11-05

**Authors:** Tânia Moniz, Sofia A. Costa Lima, Salette Reis

**Affiliations:** LAQV, REQUIMTE, Departamento de Ciências Químicas, Faculdade de Farmácia, Universidade do Porto, Portugal, Rua de Jorge Viterbo Ferreira, 228, 4050-313 Porto, Portugal; tmoniz@ff.up.pt (T.M.); shreis@ff.up.pt (S.R.)

**Keywords:** histology, permeation, pig ear skin, skin mimetic models

## Abstract

*Stratum corneum* (*SC*) represents the outermost layer of the skin, being an effective barrier against the entry of molecules and pathogens. Skin research has given particular focus to *SC* as it hampers effective drug delivery for cosmetical and therapeutical purposes. Following recommendations to develop alternative models to animals, the *SC* isolated from skin obtained from medical procedures or from pigs has gained extensive attention. Yet, there is still missing a standard and simple procedure accepted within the scientific community to avoid application of different isolated *SC* methodologies, a fact that may hamper progress in skin research. Considering this challenge, the present study evaluated different experimental conditions aiming to establish a useful and sustainable solvent-free procedure for the obtention of a realistic *SC* model. The studied trypsin digestion parameters included concentration, incubation period and temperature. Isolated *SC* was characterized using histological analysis and calcein’s permeability, after the procedure and during a 6-week storage period. Data recommend trypsin digestion at 4 °C for 20 h as the most effective procedure to isolate *SC* from pig ear skin. This work contributes to standardize the *SC* isolation procedure, and to obtain a valuable and reliable *SC* mimetic model for skin drug development.

## 1. Introduction

Transdermal drug delivery is assumed as one of the most attractive routes for administration of active ingredients. Consequently, the need to evaluate skin permeability has triggered the development of realistic skin mimetic models, mainly due to the rising of ethical questions and the establishment of new rules for human and animal tests.

To answer these challenges, many skin mimetic models have been developed, varying in complexity and mimetic properties. Different approaches have been considered, from the most simplistic non-lipid models to more complex in vitro lipid-based systems, as well as cell-based approaches, or even ex vivo human or animals skin mimetics. Some models mimic just epidermis or both dermis and epidermis, while more complex approaches include the hypodermis components and also comprise the incorporation of various skin components in the models [1].

Even though the skin is a complex organ composed by several layers [2], many *stratum corneum* (*SC*)-based models have been proposed (reviewed in [1]). These approaches are simpler than full-thickness skin mimetic models, however they represent a valuable approximation for the prediction of drugs´ permeation across the skin. This barrier represents a real challenge for the drug delivery processes due to its importance for selective permeation of drugs and protection of the skin [3,4,5,6]. Healthy *SC* plays a critical role in many physiological processes since it is the first line of contact between the environment and the body. The functions of *SC* include the prevention of excessive water loss from the skin, maintenance of body temperature, hinder the entry of xenotoxic chemicals as well as the invasion of pathogens [7]. In line with this facts, many in vitro and ex vivo models mimic mainly the *SC* since it is commonly considered the most important barrier for the study of the permeation of the drugs and thereby, understanding the drug permeation process across this layer is usually the most relevant step in drug discovery.

Due to the lipid nature of the *SC*, some in vitro models [1] considered the use of lipids or liposomes as main constituents of the mimetic membranes, respectively, known as PAMPA [8,9] (Parallel Artificial Membrane Permeability Assay) or PVPA (Phospholipid Vesicle-based Permeation Assay) [10] systems. Alternatively, another approach has been introduced, the Permeapad^®^ model [11]. In contrast to PAMPA or PVPA barriers, this model does not comprise a filter support, but it includes two cellulose membranes enclosing a layer of dry phospholipids between them. More recently, a modified version has been proposed, the Permeapad^®^ Plate, which allows permeation studies in a 96-well plate [12].

The firstly reported PAMPA system is composed by *SC* lipids [13] and later another PAMPA model has been proposed based on cellophane and *n*-octanol membranes deposited in a nitrocellulose matrix [14]. Afterwards, a skin-PAMPA system using *SC* lipid analogues has been designed and tested to inspect the permeability of different drugs [8]. Later, a modified version has also been reported [15].

Concerning PVPA models, the original system was developed to mimic intestinal barrier [16,17] but later a modified version has been designed aiming to mimic the *SC* [10]. Some other works reported the application of similar models to study the permeability of drugs [18,19,20,21,22,23]. In 2019, other two lipid-based systems have been developed [24], one with a lipid composition closer to Human *SC* (PVPA*_SC_*) [25,26].

Amongst the several reported ex vivo skin mimetic models, pig skin models are probably the most utilized due to their biological similarities with the human skin, as extensively discussed in [1]. Pig ear models have great similarities with the human skin particularly regarding the anatomical, physiological, and histological characteristics, mainly the layers’ thickness, similarity in hair follicle, blood vessel density and lipid, collagen, and elastin content of *SC*. Moreover, the permeability of pig skin was found to be similar to that of human skin, while being different from that of other animals, especially dog or rodents, as referred in [25,27]. Particularly due to the analogy with human skin layers, the central outside part of the ear is the most suitable for mimetic purposes from different parts of the pig body. Moreover, the permeability of both human and pig skin is quite similar, mainly for hydrophobic drugs (reviewed in [1]). Ex vivo *SC* mimetic models usually comprise the isolation of this layer from human or animal (pig) skin samples, however the isolation protocols are very distinct thus might hamper data correlation between publications. One of the first reports dates from 1963 [28] and describes that human skin samples are subjected to ammonia fumes or a heating at 60 °C, followed by an incubation of the skin portion, overnight, in a trypsin solution at 37 °C. Many other reports considered the isolation process of the *SC* from human or animal samples at 37 °C, using variable trypsin concentrations from 0.05 to 0.5% (*w*/*v*) [29,30,31,32,33,34,35,36]. Some of the works also refer the use of additional chemical or physical pre-treatments previous to the trypsin digestion [34,35,37], or even trypsin digestion at room temperature or at 4 °C, for long periods of time [38,39,40,41,42]. Other methods consider the isolation of *SC* keratinocytes by a two-step enzymatic digestion using dispase and type I collagenase [43] or a protein kinase inhibitor, aiming to produce a cell-based *SC* mimetic models [44]. More recently, other methods have been described, namely the so-called “tape stripping method” [45,46,47]. This procedure is considered a simple method for the evaluation of the permeability of drugs and comprises the removal of each *SC* layer by the successively use of adhesive films. In this method it is expected that each tape strip may contain the anucleate cells from the *SC* and the compound administrated in the skin, for further quantification by analytical methods [48,49]. Of note, most of these methods consider human skin and not animal skin sources, such as pig ear skin, which may present some advantages mainly since it is usually considered as a waste from the slaughters.

Due to the variety of reported isolation processes, some poorly detailed, it is crucial to standardize the isolation method and define the best experimental parameters to acquire the isolated *SC* with its typical structure and composition thus allowing the obtention of a model closer to this human skin layer. The present work focus on the solvent-free, sustainable isolation of the *SC* layer from pig ear skin using different trypsin digestion conditions, since it is one of the most accepted ex vivo skin mimetic model [26]. Evaluation of these experimental setups allowed to establish a fully detailed, valuable, and simple method for the *SC* isolation. The *SC* samples isolated using different conditions were investigated regarding their visual aspect, histological characteristics, and calcein permeability. The obtained results were discussed by comparison with other skin mimetic models, namely the full-thickness pig ear skin and the recently reported *SC* mimetic model (PVPA*_SC_*) [25].

## 2. Materials and Equipment

Porcine ears were purchased in a local slaughter (Porto, Portugal). Trypsin (from porcine pancreas), calcein and Dulbecco’s phosphate buffered saline (PBS) (10×) were purchased from Sigma-Aldrich (St. Louis, MO, USA). Double-deionized water was provided by an ultra-pure water system (Arium Pro, Sartorius AG, Gottingen, Germany). The reagents were weighted in a digital analytical balance Kern ACJ/ACS 80-4 (Kern and Sohn; Balingen, Germany). pH measurements were achieved using a JENWAY 550 pH meter (UK). Histological samples have been processed in the histological service of ICBAS, University of Porto.

## 3. Experimental Design

### 3.1. Study Aims

The main aim of the present study is the establishment of optimal set of experimental conditions to obtain a simple and effective protocol for the obtention of a reliable human *SC* mimetic model. For this, specific objectives have been defined:

i. Design and evaluation of different experimental parameters, varying the trypsin concentration, the temperature, the time of digestion and the freezing of the skin prior to digestion;
ii. Study of the influence of each set of conditions on the isolated *SC* regarding the visual aspect, histological features and permeability properties;
iii. Characterization of the *SC* model isolated in the selected optimal conditions by assessment of the histological morphology and storage stability.

### 3.2. Preliminary Steps: Preparation of Pig Ear Skin

The process of *SC* isolation started by the preparation of pig ear skin. The porcine ears were obtained from different animals and were purchased in a local slaughter (Porto, Portugal). Initially the ears were cleaned with tap water and dried with soft paper. If needed, remaining hairs were removed with the help of a regular shaving blade. Then the external part of the pig ear skin was removed from the underlying cartilage using a scalpel. Only the central outside part of the ear has been utilized. The subcutaneous fat tissue was carefully removed, thus ensuring the integrity of the skin. The areas presenting visible capillary veins or marked with stamps have been discarded.

### 3.3. Design of the Methodologies and Visual Inspection of the Skin after Trypsin Digestion

The experimental conditions chosen for the trypsin enzymatic digestion can influence the efficacy of the process thus altering the integrity of the *SC* and consequently the permeation of the drugs across the skin layer.

**Figure 1:**
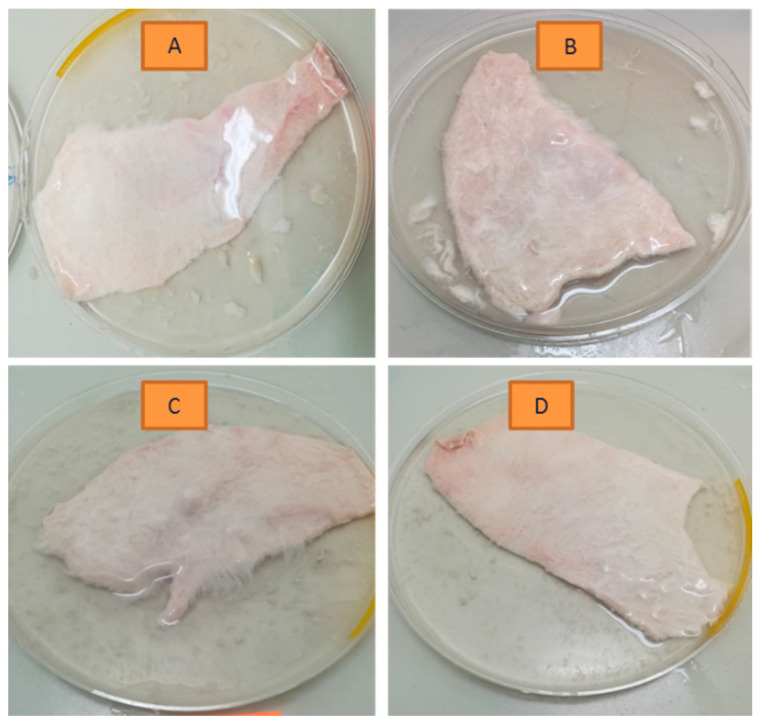
Visual aspect of the skin portions subjected to the different experimental conditions, after the defined incubation time. (**A**–**D**) conditions are detailed in Table 1.

**Figure 2:**
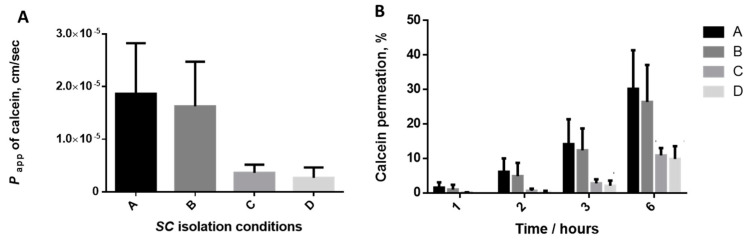
Effect of *SC* isolation conditions on the permeability of calcein through the pig ear *SC*. (**A**) Apparent permeability coefficient at 3 h; (**B**) Percentage of permeation of calcein at different timepoints. Error bars represent the mean ± SD for at least three independent experiments. *p* < 0.05 for Kruskal-Wallis test among the studied *SC* isolation conditions. Black, dark grey, medium grey and light grey bars correspond to isolation conditions A, B, C and D, respectively.

**Figure 3:**
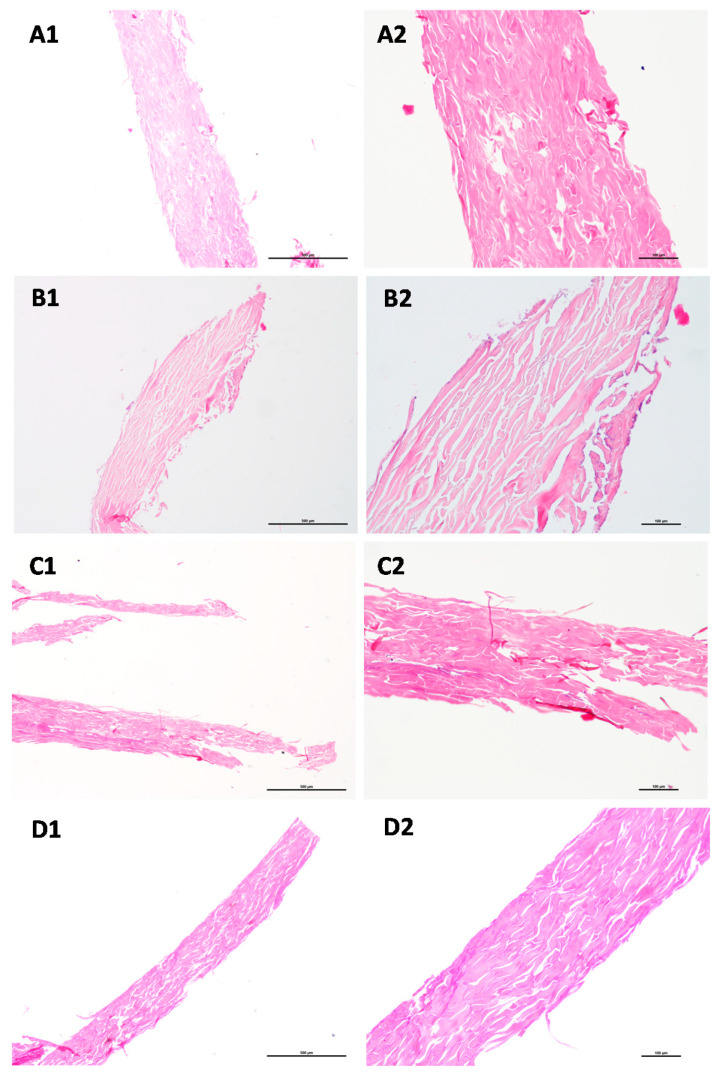
Histological appearance of *SC* after exposure to different isolation conditions ((**A**–**D**), according to the designations defined in Table 1). HE staining; Scale: (**A1**–**D1**) (4×), bar 500 µm; (**A2**–**D2**) (10×), bar 100 µm.

Thus, different conditions were tested varying the: (a) time of digestion; (b) temperature of incubation; (c) concentration of the trypsin solution; and (d) pre-freezing of the skin. All conditions (A–D, Table 1) have been studied based on (i) visual observations (Figure 1); (ii) calcein permeation through the isolated *SC* (Figure 2); and (iii) histological characterization (Figure 3).

Based on Becker studies [42], the routinely used protocol in the lab involves the trypsin digestion of the skin sections at 4 °C [26,50]. The adopted procedure considers skin obtained from pig ear and excludes the final step of incubation at 37 °C as well as the use of trypsin inhibitor. In addition, the trypsin solution was renewed after the first 4 h of incubation at 4 °C. These parameters were considered as the reference condition (Condition A, Table 1). Additionally, other set of conditions were studied, namely the effect of a pre-freezing of the skin, since it is common to receive pig ears from slaughter houses, isolate the external skin portion and freeze it until the skin is required for studies (for a maximum of 2 months). Thus, a set of conditions was defined in which the skin portion was frozen for 48 h prior to the trypsin digestion (Condition B, Table 1). Before the immersion in the trypsin solution for digestion, the skin was let to totally defrost at room temperature. Temperature and time of trypsin digestion was maintained equally to the set of conditions A (Table 1).

For the optimization of the isolation, a 0.1% and 0.05% (*w*/*v*) trypsin (Sigma-Aldrich, St. Louis, MO, USA) solution in phosphate-buffered saline (PBS, 1×) (diluted from PBS 10×, Sigma-Aldrich, St. Louis, MO, USA) was prepared and the pH adjusted to 7.4. Then, the isolated pig ear skin was placed in a plastic Petri dish (Tissue Culture Dish 150, 147.8 cm^2^, TPP), placing the internal part facing the bottom of the dish, allowing the skin to be immersed in enough trypsin solution (0.1% or 0.05% (*w*/*v*), Table 1) to cover the tissue. The Petri dish was incubated at the selected temperature (4 or 37 °C, Table 1). During the incubation time, at defined intervals of approximately 1 h, the skin was removed from the Petri dish and it was scraped with the help of a scalpel, to detach the viable epidermis and the dermis from the *SC*. For experimental condition A and B (Table 1), the trypsin solution was replaced with fresh solution for a better digestion and the skin was let to incubate overnight at 4 °C.

The other experiments considered the incubation of the skin with the trypsin at 37 °C for 3 h (Condition C, Table 1). Those conditions were selected as many reports in the literature described the use of similar experimental conditions, particularly the digestion at 37 °C [29,30,31,32,33,34]. As the digestion process is based on an enzymatic digestion and usually the optimal temperature is around physiological values, a temperature of 37 °C was considered to increase the yield of the enzymatic reaction. Keeping the latter conditions, the concentration of the trypsin solution was decreased to 0.05% (*w*/*v*) since it is expected that at higher temperature values, the digestion may be fasten, the efficacy of the process may be increased and therefore the amount of enzyme needed can be diminished (Condition D, Table 1).

After the total digestion period, the *SC* was rinsed with ultrapure water to stop trypsin digestion and the remaining dermis tissue was removed with the help of a scalpel.

**Figure 4:**
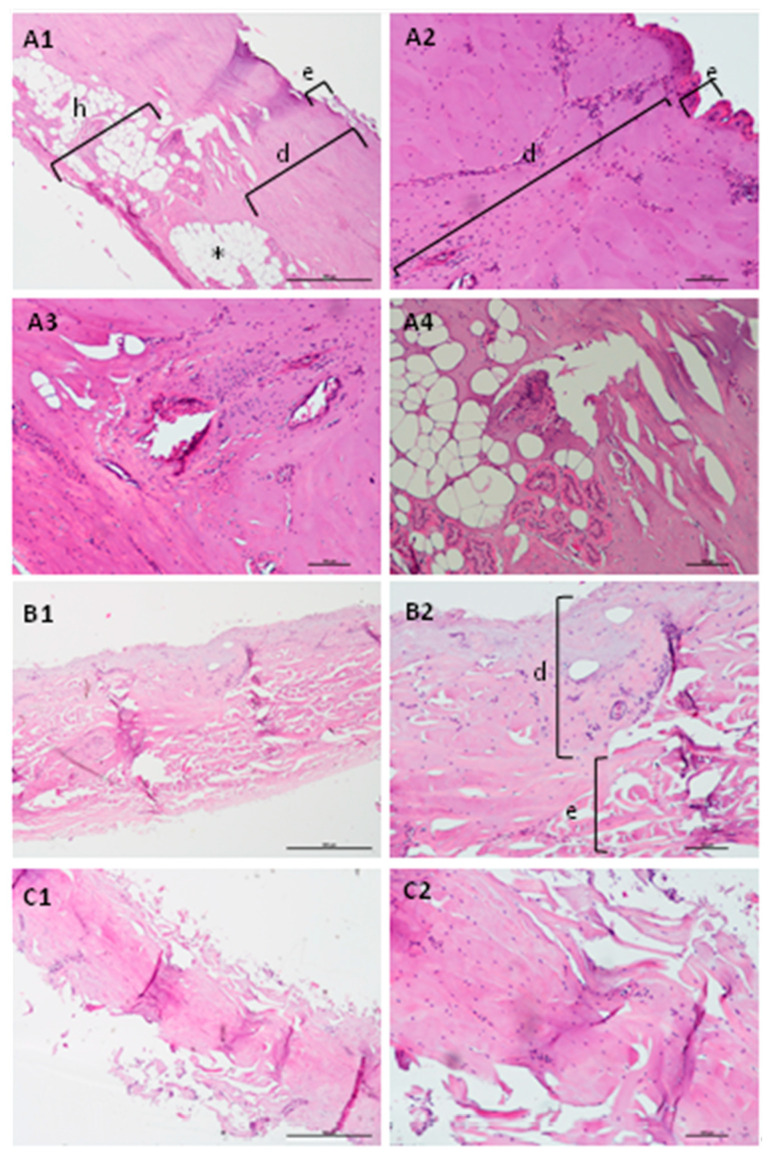
Histological sections of skin portions collected during the *SC* isolation process. (**A**)—samples collected prior to trypsin digestion (0.1% *w*/*v*) (**A1**–**A4**); (**B**)—after 4 h incubation at 4 °C; (**C**)—after overnight digestion. Asterisks represent vacuoles derived from adipocytes; d—dermis; e—epidermis; h-hipodermis. HE staining; Scale: (**A1**–**C1**) (4×), bar 500 µm; (**A2**–**A4**,**B2**,**C2**) (10×), bar 100 µm.

At the end of each defined incubation time, the visual aspect of the skin portions subjected to the different experimental conditions was registered in photography as summarized in Figure 1A–D. It was possible to understand by the handling of the skin portions and by the visual observation (Figure 1A–D) that the skin portions subjected to conditions A and B presented less amount of residues from dermis and the cleaning process was easier since the dermis has been detached better from the epidermis than from the skin subjected to experimental conditions C and D. These findings pointed out the importance of the temperature during the isolation process and revealed that the incubation of the skin portions at 4 °C for a longer period of time seems to be advantageous. Of note, preliminary tests have been made considering the incubation of the skin portions at 4 °C for a shorter period, particularly 3–4 h, however it was possible to understand by visual observation (data not shown) that these conditions were not enough for the dermis to be totally detached. This point was also confirmed in the subsequent studies, as depicted in Figure 4(B1,B2). Considering this, conditions A and B included the replacement of the trypsin solution after 4 h of incubation at 4 °C and the following overnight incubation.

Comparing conditions A and B, no major differences were detectable with the naked eye and by the handling in the *SC* isolation process, a fact that suggests that the pre-freezing of the skin does not influence the isolation process of the *SC* layer. For this reason, pre-freezing of the skin was not considered for the following studies of trypsin digestion at 37 °C.

Regarding conditions C and D, for identical time and incubation temperature, the concentration of the trypsin solution seems not to be relevant within the studied range, since the appearance of the skin was quite similar and the detaching of the dermis occurred in a similar way.

The slower rate of digestion conferred at 4 °C (conditions A and B) allowed a better control of the ideal point to stop the process. The reactions at 37 °C (conditions C and D) are quicker but, if the process is not stopped in the adequate timepoint, it may conduce to a scenario of excessive digestion of the skin layers.

Finally, the *SC* was left to dry in a silica-containing desiccator at atmospheric pressure until completely dried. This process occurred up to one week.

The different conditions selected during the optimization of the procedure, particularly regarding trypsin concentration, temperature of the incubation process and pre-freezing of the skin, are summarized in Table 1.

### 3.4. Evaluation of the Calcein Permeability

The integrity of the *SC* layer isolated in each set of conditions (Table 1) was evaluated using a calcein permeation assay. Calcein was taken as a model compound [25,26]. The permeation profile of this molecule has been already documented for other skin models [10,19,20,25,26], mainly the full skin pig ear and the PVPA*_SC_* mimetic systems and these findings allow us to compare the data obtain herein with those previously reported.

The permeation studies were performed according to the permeability studies using full-thickness pig ear skin [25]. Briefly, the dried isolated *SC* portions from the 4 studied conditions were cut in circles (approximately 2.5 cm diameter) which were then placed between donor and acceptor compartments of Franz diffusion cells (9 mm unjacketed Franz Diffusion Cell with 5 mL receptor, O-ring joint, clear glass, clamp and stir-bar; PermeGear, Inc., Hellertown, PA, USA) [51]. The acceptor chamber was filled with 4.7 mL of PBS (pH 7.4) and the calcein (Sigma-Aldrich, St. Louis, MO, USA) solution (3.11 × 10^−4^ g, 0.5 mL of 1 mM solution in PBS, pH7.4) was added to the donor compartment, covered with parafilm. During the study, the Franz cells were stirred, maintained at 37 °C and protected from the light. Aliquots of 0.2 mL were collected from the acceptor chamber at the timepoints 1, 2, 3 and 6 h. The quantification of the calcein concentration in all collected samples was estimated by the apparent permeability (*P_app_*) (3 h) which was determined accordingly to the following equation [25]:

(1) $$P_{app}\left(\mathrm{cm}/s\right)=\frac{\sum m_{a}}{m_{d}\cdot A\cdot t}$$

The *P_app_* was estimated by the ratio of the sum of the mass of calcein (*m_a_*/g) permeated across the *SC* and the product of the initial mass in donor chamber (*m_d_*/g), the diffusion area between cells in Franz diffusion system (0.63585 cm^2^) and the time (3 h = 10,800 s).

Permeation results were analysed using GraphPad Prism Software (Version 6.0 for Windows; GraphPad Software Inc., San Diego, CA, USA). The results are expressed as mean ± SD for at least three independent experiments. Levene’s and Shapiro-Wilk tests showed that data is not normally distributed. The nonparametric Kruskal Wallis test was perform and significance was accepted when *p* value < 0.05 was obtained.

The apparent permeability of calcein at 3 h (Figure 2A) through the *SC* isolated in experimental conditions A and B is higher than those obtained for *SC* isolated in condition*s* C and D. Two subsets of results were obtained as no big differences were found between conditions A vs. B or also comparing *SC* isolated in condition*s* C *vs.* D, which correlate with the temperature of the trypsin digestion. Comparing the *P_app_* values of calcein through the isolated *SC* in different conditions and those previously obtained in PVPA*_SC_* model, the values determined for conditions A and B are more similar than those obtained in condition*s* C and D. These facts pointed out that conditions A and B should be preferred for the isolation of the *SC* as they allow the obtention of values in accordance with the expected magnitude for this skin layer (10^−5^ cm·s^−1^) [26] and distinct from the values reported for an ex vivo full-thickness skin pig ear model (10^−6^ cm·s^−1^) [25]. The obtained values for the calcein permeation at 3 h regarding the *SC* portions obtained in condition*s* C and D are in the same magnitude order of those previously reported for the full skin pig ear model (10^−6^ cm·s^−1^) [25]. These facts may evidence that the digestion process was not so effective than the verified for conditions A and B. The percentage of calcein permeated along the time (Figure 2B) confirms the differences between the samples obtained by conditions C and D vs. the *SC* isolated in conditions A and B. 

The comparison of the permeability of the *SC* obtained by the isolation methods herein discussed with other ex vivo *SC* mimetic models isolated by other methodologies described in the literature [28,29,30,31,32,33,34,35,36,37,38,39,40,41,42] is not possible since the alternatives approaches mainly focus on analysis of the structural features and lipid composition of the isolated models and do not deeply explored the permeation profile and the integrity of the obtained layers. Few studies explored the permeation of other molecules, but no information has been reported regarding calcein permeation [31,38,40].

### 3.5. Histological Characterization

To proceed with the selection of optimal experimental conditions for the *SC* isolation, the histological analysis has been performed. Samples from the *SC* layer isolated from pig ear skin in different experimental conditions have been collected at two stages: (i) immediately after stopping the digestion and, (ii) after the drying process. All the samples were processed routinely and embedded in paraffin wax. Sections were stained with haematoxylin and eosin (HE) for histology analysis [52,53]. The analysis was performed by light microscopy and representative images are depicted in Figure 3.

Data demonstrated that the appearance of the *SC* layer obtained in each set of experimental conditions (A–D, Table 1) is similar (Figure 3). For the representative images displayed in Figure 3, the thickness of the section was estimated and for conditions A and B the *SC* layer has 434 ± 7 µm and 497 ± 13 µm, respectively. However, the samples obtained for the isolation under conditions C and D are thinner (274 ± 13 µm and 290 ± 17 µm, respectively) than those obtained for digestion process considering the experimental set of conditions A and B. This information can explain the distinct permeability values of the *SC* models obtained in conditions A/B vs. C/D, since the thinner layer may be more compact and therefore less permeable.

For all the conditions studied, it was possible to verify that after the exposure to the trypsin digestion treatment *SC* sheets remain intact and the subjacent layer, particularly the *stratum granulosum,* was complete removed. There was no evidence of any portion of dermis in the sections analysed. In addition, no nucleated cell layers usually found on the epidermis were possible to be detect, assuring complete removal of this viable layer from the isolated *SC*. Only the corneocytes sheets are present in *SC* and the empty spaces observed between the layers may be due to the lipid’s removal upon the preparation of the histological sections.

## 4. Results

### 4.1. Characterization of the Model Obtained in the Selected Conditions: Histological Analysis

Despite the fact that no major differences have been detected in the morphology of the *SC* layer isolated considering the different experimental conditions, the permeation results described above evidenced that condition A represents the most advantageous set of parameters to obtain a realistic *SC* mimetic model. Thus, this condition was chosen for the routine *SC* isolation and was further characterized.

To better understand the transformation that occurred in the pig ear skin during the chosen *SC* isolation protocol (condition A, Table 1), fresh skin portions have been collected in different timepoints of the procedure for subsequent histological analysis. Samples have been collected previously to the trypsin digestion process (Figure 4A1–A4). In this timepoint, it is possible to identify the three major layers of the skin, epidermis, dermis and hypodermis (e, d and h, respectively, in Figure 4A1)), thus pointing out that the skin remains intact after the procedure for the detachment of the external part of the skin from the pig ear. It is also possible to observe empty vacuoles derived from adipocytes, which are destroyed during the processing of the samples for histological analysis due to their lipid nature (assigned with asterisks, Figure 4A1,A4.

After 4 h of trypsin digestion at 4 °C, another portion of the skin and the histological analysis shown in Figure 4B1,B2, where is evident a significative reduction on the complexity of the skin structure. Some layers have been digested at this time: no evidence of hypoderm is present, the dermis layer revealed some signals of disintegration (d, Figure 4B2), while the epidermis (e, Figure 4B2) remains intact.

After an overnight incubation with trypsin, the histological analysis revealed the depletion of the dermis as well as part of the epidermis and uniquely the presence of the *SC* layer can be visualized. However, some nucleated cells (stained as purple dots, Figure 4C1,C2) are still present at this timepoint. It is important to mention that this section has been analysed prior to the drying process required as the last step of the *SC* isolation process. As illustrated in Figure 3A1,A2, after the drying of the skin portions, no evidence of nucleated cells has been detected. Clearly some alterations have been occurred during the drying step, which is imperative for the obtention for the *SC* mimetic model and their use in applications, such as permeation studies.

### 4.2. Characterization of the Model Obtained in the Selected Conditions Storage Stability

To study the stability of the *SC* along the time and to define the ideal duration of the drying process, the *SC* isolated under condition A were left to dry in a desiccator at room temperature and atmospheric pressure. Since the isolated *SC* was complete dried, fragments were collected, and the permeability was accessed as described above. The isolated *SC* were kept up to 6 weeks in the desiccator under above defined conditions. At defined timepoints (1, 2, 4 and 6 weeks), calcein permeation through the isolated *SC* was examined, as described above.

**Figure 5:**
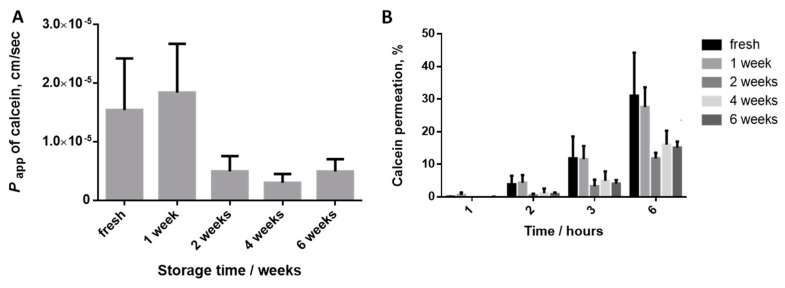
Effect of the drying time of the isolated *SC* on the permeability of calcein. (**A**) Apparent permeability coefficient at 3 h; (**B**) Percentage of permeation of calcein at different timepoints. Error bars represent the mean ± SD for at least three independent experiments. *p* < 0.05 for Kruskal-Wallis test among the storage time.

Results obtained have shown that the permeation profile of calcein is similar for isolated *SC* dried for the minimum time (first time complete dried, assigned as “fresh”, Figure 5A) and after one week. These values are in the same magnitude of the values found for the PVPA*_SC_* mimetic model (10^−5^ cm·s^−1^) [25,26] and distinct from the values reported for the ex-vivo full-thickness skin pig ear model (10^−6^ cm·s^−1^) [25]. After two additional weeks of drying, the *P_app_* of calcein clearly decreased to values in the same magnitude of the previously found for the ex vivo full-thickness skin pig ear model (10^−6^ cm·s^−1^) (Figure 5A). After 4 or 6 more weeks of drying, the calcein permeation significantly decreased and reached values characteristics of the whole pig ear skin [25] (Figure 5A). The analysis of the results depicted in Figure 5B confirmed that after two additional weeks in the drying process, the percentage of permeability of calcein clearly decreased when compared to fresh condition. This evidence is notable for drug permeated after 2 h, and even more declared at 3 and 6 h of permeation. Accordingly, it seems possible to conclude that in order to acquire a *SC* model with permeability profile characteristic of this layer, the isolated *SC* can remain just one more week in the desiccator after completely dried (fresh condition). After this time, the drying process should be stopped by maintaining the *SC* portions in a Petri dish sealed with Parafilm and stored at room temperature and pressure. The excessive loss of water expected for higher periods of drying influenced the integrity of the model and subsequently the decrease in the permeability of hydrophilic calcein.

## 5. Limitations

The herein proposed methodology does not include the apocrine pore/skin hair holes assessment. Therefore, the permeation results obtained considering *SC* mimetic samples prepared by the present method will not allow the acquire insight on the origin of the drugs’ diffusion. Thus, it must be considered that the permeation may be caused by the self-diffusion of the drugs across the skin layer, by passage across porous/hair skin holes, or by the combination of both events.

## 6. Concluding Remarks

Among the different *SC* isolation procedures for the obtention of mimetic models, the inconsistency of experimental conditions is vast, thus leading to a great variability of results which difficult the comparison among studies performed using different isolation strategies. Moreover, some of the already described methods are in general poorly detailed regarding the experimental conditions or use non-benign solvents and non-sustainable isolation conditions [28] as well as the requirement of additional chemical or physical treatments previously or posteriorly to trypsin digestion.

**Figure 6:**
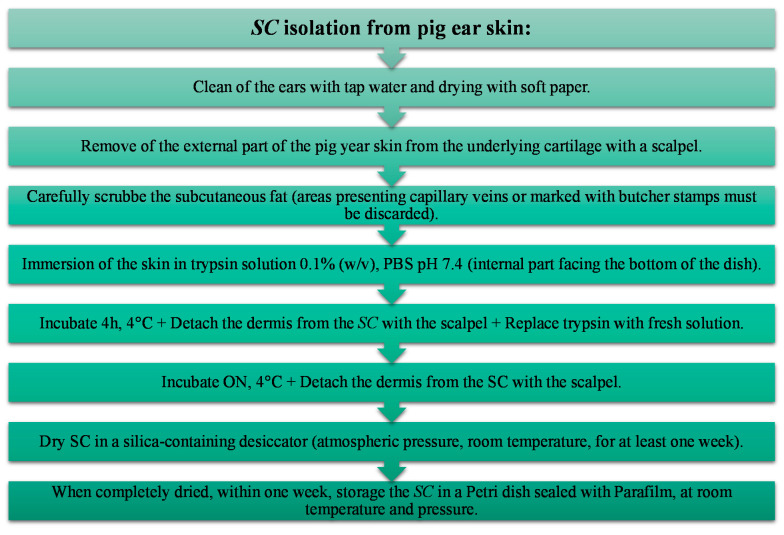
Flow chart of the protocol defined for *SC* isolation from pig ear skin by trypsin digestion.

The present study was determinant to understand the effect of different experimental conditions and to establish the optimal procedure for the obtention of a realistic *SC* model from pig ear skin. Considering the integrity of the skin layer as well as the histological the optimal conditions for the *SC* isolation involve skin trypsin digestion (0.1% *w*/*v*), at 4 °C, during approximately 20 h, as summarized in the flow chart represented in Figure 6.

The permeability assay pointed out that this slower digestion process at low temperature is valuable for the obtention of a mimetic model with permeability characteristics usually found for other *SC* mimetic models, mainly PVPA*_SC_* barriers. The isolated *SC* must be stored in proper conditions after being completely dried to maintain its permeability and integrity properties.

At the end, it was possible to establish a simple procedure for *SC* isolation from pig ear skin, by trypsin digestion at low temperatures, followed by drying under controlled conditions. Moreover, in the herein described methodology the number of steps has been reduced when compared to other methods previously described, namely by avoiding the use of additional reagents such as trypsin inhibitors. Thus, it was possible to establish a quicker, less expensive and, more sustainable approach for SC isolation from pig ear skin. The wide implementation of the herein described protocol will allow the possible comparison of results obtained by distinct investigations.

## Figures and Tables

**Table 1:** Experimental conditions considered for the trypsin digestion of the pig ear skin to obtain *SC* layer.

Condition	Incubation Time	Temperature	Trypsin Concentration	Pre- Freezing
A	4 h + O/N ^1^ (total ~ 20 h)	4 °C	0.1%	No
B	4 h + O/N ^1^ (total ~ 20 h)	4 °C	0.1%	Yes, 48 h
C	3 h	37 °C	0.1%	No
D	3 h	37 °C	0.05%	No

^1^ O/N—overnight.
